# Model Test Study on the Enhancement of Ecological Self-Repairing Ability of Surface Slope Soil by New Polymer Composites

**DOI:** 10.3390/ijerph19169933

**Published:** 2022-08-11

**Authors:** Wei Huang, Cuiying Zhou, Zhen Liu

**Affiliations:** 1 School of Civil Engineering, Sun Yat-sen University, Guangzhou 510275, China; 2 Guangdong Engineering Research Centre for Major Infrastructure Safety, Sun Yat-sen University, Guangzhou 510275, China

**Keywords:** soil improvement, slope protection, model test, long-term effect, ecological self-repairing

## Abstract

Plant-based ecological protection is one of the effective methods to improve the stability of slope soils. However, plants need a stable growth environment and water supply. Although it has been demonstrated that polymer materials can effectively enhance the stability and water retention of soils, their improvement mechanism and long-term effects are yet to be clear. In this paper, we use a new polymer composite material (ADNB), an optimized compound of nano-aqueous binder (NAB) and super absorption resin (SAR), to conduct outdoor model tests to study the effects of different ADNB ratios on soil compactness, biochemical properties, and plant growth at longer time scales, and to explore its action law and mechanism of enhancing the ecological self-repairing ability of surface slope soil. The results show that ADNB can effectively improve the soil structure, increase the compactness of the soil, increase the organic matter content, microbial population and available nutrient content in the soil, thus promoting plant growth. The adsorption and agglomeration effect of the NAB in ADNB on soil particles and its degradation in natural environment can be observed by SEM. In summary, ADNB can not only effectively enhance the ecological self-repairing ability of surface slope soil, but also has good environmental friendliness and can be completely degraded under natural conditions without additional adverse effects on soil and environment.

## 1. Introduction

The newly excavated soil slope has loose structure and low mechanical strength. It is easy to be damaged by rainfall erosion, causing soil erosion and serious destabilization of the slope, threatening the safety of nearby buildings [1]. The traditional slope protection technology mainly focuses on engineering protection, ignoring the active role of plants in soil and water conservation and ecological restoration. Recent studies have shown that the roots and leaves of plants have excellent performance in improving soil strength and reducing erosion [2,3,4], and complete vegetation cover can reduce slope erosion by more than 80% [5]. Therefore, a higher vegetation cover is the key to prevent soil erosion on slopes. However, it is still a challenge to ensure good plant growth, especially how to improve the germination rate of plant seeds and survival rate of seedlings before the vegetation communities that can naturally evolve are fully formed.

Stable environment and adequate water supply are essential for seed germination and seedling growth. To provide a stable soil environment, curing agents have been used to reinforce the soil [6]. Currently, the common curing materials are divided into three categories: inorganic, organic, and biological. Inorganic materials mainly include cement, gypsum, and fly ash [7], which have the advantages of short consolidation time and high mechanical strength, but most inorganic materials have strong acid/alkaline or excessive heavy metal ions, which can pose a threat to plant growth. Organic materials are diverse and have different properties, their controlled material composition makes them generally environmentally friendly, and they are widely used in slope ecological protection [8,9]. Biological materials are generally bacteria [10], which use their metabolism to produce urea that combines with metal cations to produce gelling crystals, which have the effect of reinforcing the soil. This technique is environmentally friendly and cures well. However, its disadvantages are the long curing time and the complexity of the operation, which makes it unsuitable for use in slope ecological protection projects. Polymer curing agents, as a type of organic materials, have triggered a lot of attention from scholars due to their lower production cost and excellent adhesion properties [11]. In the process of soil improvement, the high viscosity of polymer curing agent can not only improve the soil structure, enhance the soil strength, and reduce the soil compressibility [12] but also increase the content of soil agglomerates and improve the water stability of the agglomerates [13]. The stability of the topsoil is improved by forming a hardened layer on the surface, thus improving the soil and water conservation and ecological restoration effect of the slope; relevant studies have shown that polymeric curing agents can also effectively inhibit the expansion of swelling soils [14] and increase the proportion of easily oxidized organic carbon in the soil [15].

In order to improve the water supply capacity of soils, superabsorbent resins have been introduced [16]. Its special three-dimensional-like molecular chain structure allows it to absorb and conserve large amounts of water in a short time [17]. Once the water content of the soil is low, the water stored in the resin is gradually released by the suction of the substrate and supplied to the plant growth, thus alleviating the drought stress [16]. Studies have shown that, on the one hand, the resin improves soil fertility by increasing the ability to retain water and hold nutrients [18]. On the other hand, the resin loosens the soil by volume changes during water absorption and release, thus creating beneficial conditions for water and nutrient transport [19]. Therefore, the addition of resin to the soil can effectively optimize the water and nutrient transport pattern of the soil and promote plant growth; in addition, related studies have shown that the addition of resin can effectively reduce the soil bulk, improve the stability of soil aggregates, indirectly promote microbial reproduction, and increase the diversity of bacterial communities [10].

In summary, curing agents and resins can effectively improve the physical, chemical, and biological properties of soils to promote plant growth. However, both types of materials have their own shortcomings. Although curing agents can strengthen the soil structure and improve the erosion resistance of slopes, their water retention properties are poor and cannot increase the survival rate of plants under drought conditions; on the contrary, resin materials, although they can significantly increase the water content of the soil, the volume change in the process of water absorption and release will destroy the original structure of the soil skeleton and lead to a decrease in slope stability. Therefore, to address the respective advantages and disadvantages of the two materials, researchers at Sun Yat-sen University developed a new polymer composite material (ADNB) using an optimized compounding of polymer curing agent and resin to improve slope stability and water supply using a bio-chemical synergistic approach. Through the preliminary indoor tests [20] and field tests [21], ADNB has shown good soil and water conservation and ecological restoration effects. However, the scale of indoor tests is small and the amount of information that can be obtained is limited, which is not conducive to in-depth research. The field test has too many uncontrollable factors due to the randomness of stratigraphy, lithology, and groundwater distribution, and the method is only applicable to the study of macroscopic parameters, but the accuracy of the data cannot be guaranteed for microscopic parameters which are influenced by the environment (e.g., microorganisms, soil nutrients, organic matter distribution, etc.). It is often necessary to use the average of a large amount of data to avoid the bias of the conclusion caused by the local area dispersion. In field projects, it is often difficult to find a test area that meets the demand. Therefore, this paper uses outdoor model tests to control for non-essential variables and obtain accurate results for microscopic parameters with a smaller amount of data.

This paper investigates the effects of different ADNB ratios on soil compactness, biochemical properties, and plant growth at longer time scales through outdoor model tests, and explores its effects on enhancing the ecological self-repairing ability of surface slope soil.

## 2. Climatic Conditions in the Study Area

**Figure 1:**
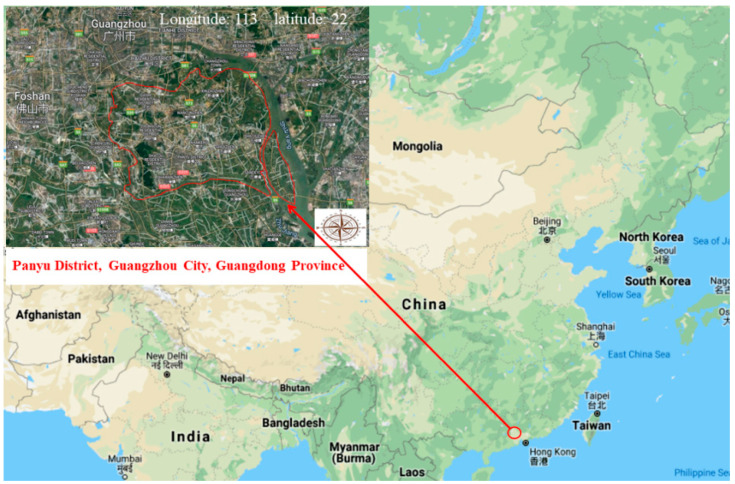
Geographical location of the study area.

The Panyu District of Guangzhou City is located in South China (113 degrees east longitude, 22 degrees north latitude) (Figure 1). It belongs to the south subtropical marine monsoon climate, characterized by warmth and rain, sufficient light, and a long frost-free period. The average annual precipitation is 1633 mm, the average annual evaporation is 1670 mm, the average annual temperature is 22.1 °C, and the average annual sunshine hours is 1472 h.

**Figure 2:**
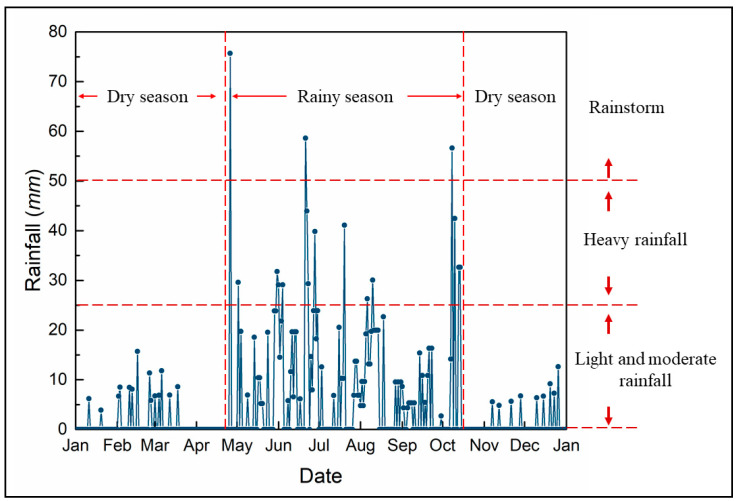
Rainfall data in the study area (National Meteorological Information Center).

According to the rainfall data of the National Meteorological Information Center in 2021 (Figure 2), it can be seen that the rainfall in the study area is rich but unevenly distributed during the year. The rainy season is from mid-late April to mid-October, accounting for 88.93% of the total annual rainfall. Among them, April to June is mostly the southwest monsoon, which contains a large amount of vapor. When it meets the cold air falling south, it often leads to heavy rainfall. The southeast monsoon prevails from July to October, and the thermal cyclones in the Pacific and South China Sea bring large amounts of vapor, forming strong wind and rainstorm. Northeast winds prevailed from mid-October to March of the following year, mostly in dry season, and rainfall only accounted for 11.07% of the total annual amount.

## 3. Test Materials and Methods

### 3.1. Materials

Test materials include test soil and new polymer composites (ADNB).

The test soil was collected from the Panyu District, Guangzhou City, Guangdong Province. It is a common silty clay in South China. It is a weathering product of Cretaceous clastic rocks, Yanshanian granite, and Proterozoic metamorphic rocks. The physical and mechanical parameters of soil are shown in Table 1. Soil sample after air drying, crushing through 2 mm sieve spare.

The new polymer composite (ADNB) mainly includes nano-aqueous binder (NAB) and super absorption resin (SAR). The NAB is mainly composed of polyvinyl acetate (Polyvinyl Acetate), and the molecular formula is [CH_2_CHCOOCH_3_]_n_. Its appearance is milky white liquid, its pH value is 6–7, its density is 1.01 g/cm^3^, its solid content is 41%, its viscosity is 8000–10,000 mPa s, and it is insoluble in water. However, it has good dispersion in water and can be configured into aqueous solution. Under natural conditions, it can be slowly degraded. The degradation period is 24 months, and the final products are CO_2_ and H_2_O. The main component of the SAR is sodium polyacrylate, and the molecular formula is C_3_H_3_NaO_2_. At room temperature, the SAR is white particles with particle size ≤ 0.02 mm, moisture content ≤ 5%, and bulk density 0.8–0.85 g/mL. After saturation, the SAR is transparent hydrogel, and the theoretical water absorption is about 250%.

According to the previous studies [20,21], the NAB dosage in Guangzhou area is 10–15 g/m^2^, and the SAR dosage is 60–70 g/m^2^, which has good soil and water conservation and ecological restoration effect. Therefore, this paper uses the ratio shown in Table 2 to carry out model tests to study the long-term repair effect of ADNB on soil.

### 3.2. Test Method

A uniform mixture of different doses of SAR was mixed with soil and then loaded into plastic boxes (length × width × height: 60 cm × 45 cm × 30 cm) with a soil layer thickness of 20 cm (the soil in each box weighs about 92 kg), using the layered filling method to ensure that the initial density of the test soil was all 1.7 g/cm^3^. Then, the water-soluble NAB was evenly sprayed on the soil surface. In order to simulate the engineering slope more truly and keep the test box inclined, the slope is 1:1.15 common in slope design. Total duration of the test is one year (start and end dates: 31 December 2020–31 December 2021).

#### 3.2.1. Compactness of Soil

In situ measurement is carried out by using a soil compactness meter (penetrometer) produced by the STEP company in Germany [22]. The basic principle is to characterize the soil compactness by measuring the insertion resistance of the front probe. Ten points were evenly selected on the surface of the soil sample, the compactness values of the soil at these points were measured successively, and the average value was taken. When measuring, the compactness instrument should be perpendicular to the slope surface to avoid the front end of the probe with hard or root block.

#### 3.2.2. Biochemical Properties of Soil

The biochemical properties of the soil were measured once per quarter. Soil samples were collected at 6 points evenly selected on the surface of the soil samples, each soil sample was tested, the results were averaged, and the standard deviation of the data was calculated. The collection time was the 15th day of the middle month of each quarter. In total, the samples were sampled four times: on the 15th of February, 15th of May, 15th of August, and 15th of November, in that order. Since total soil nutrients (total *n*, *p*, and K) reflect the total amount of nutrients in the soil, which need to be converted into fast-acting nutrients to be absorbed by plants and are not directly related to plant growth, they were only measured at the beginning of the test (1 week after the start of the test), which was used to calibrate the base nutrient status of the soil.

Soil organic matter using potassium dichromate capacity method [23]; soil total nitrogen using the semi-micro Kjeldahl method [24]; soil total phosphorus using spectrophotometry [25]; soil total potassium using flame photometer [26]; soil-available nitrogen by alkaline hydrolysis diffusion method [27]; soil-available phosphorus using the NaHCO_3_ extraction method [28]; and soil-available potassium were extracted with neutral ammonium acetate solution and determined by flame photometer [29]. Nutritional agar for bacteria [30]; potato glucose agar medium (PDA) for fungi [31]; and actinomycetes were cultured in Gaoshi No.1 medium [32].

#### 3.2.3. Plant Growth

The common slope-protecting plant in South China was selected as the test object (*Crotalaria pallida*). Before the test, the seeds were soaked in water for 12 h, and the dry and wilted seeds were removed manually. Seeds were evenly spread to the surface soil, and the number of seeds was 100. The germination rate and germination time were manually counted, the results were averaged, and plant height was recorded periodically.

This test unified water and fertilizer management. According to the rainfall data (Figure 2), it can be seen that the rainy season is from April to September, and the dry season is from January to April and after mid-October each year. Therefore, according to the actual project, artificial irrigation is used to simulate rainfall, and rainfall is 5 mm/day, to ensure seed germination and growth of plants. By the middle of April, after entering the rainy season, artificial irrigation is halted until the end of the experiment, and soil moisture is completely supplemented by natural rainfall. In order to simulate the real state of plants in the slope ecological protection project, although it entered the dry season after mid-October, it did not carry out artificial irrigation, still relying on natural rainfall recharge until the natural apoptosis of plants.

## 4. Result

### 4.1. Variation Trend of Soil Compactness

**Figure 3:**
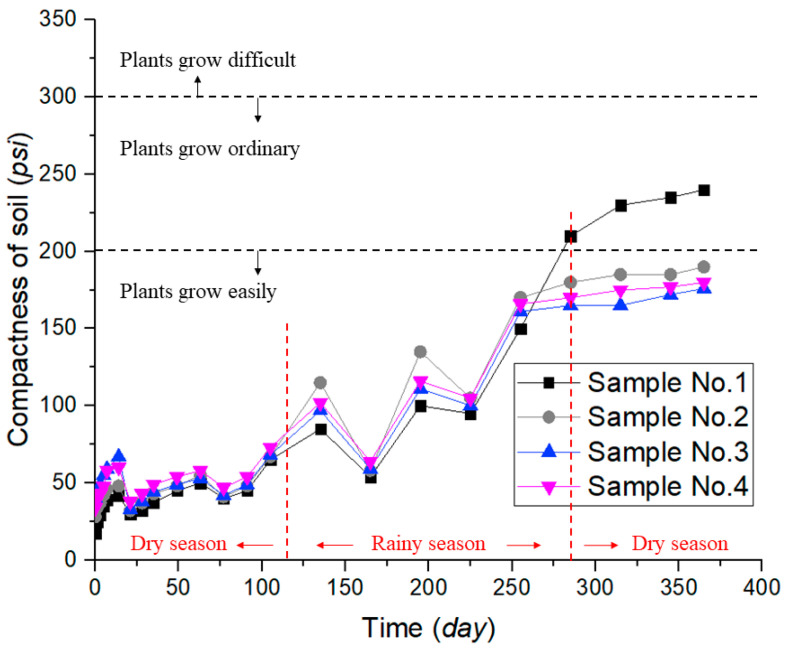
Compactness of soil.

Soil compaction is a measure of the ability of a soil to resist compaction and crushing by external forces and is measured in pounds of force per square inch (*psi*). It can be seen from Figure 3 that the compactness of the soils was small at the beginning of the test (0–110 days), and with the help of ADNB, the compactness of the test group (No. 2–4) was slightly higher than that of the CK (No. 1) (increased by 6–135.3%). When the rainy season came (110–285 days), the repeated wet and dry cycles and rainfall scouring led to the gradual hardening of the CK (No. 1) soil and increasing compactness. After the end of the rainy season (285–365 days), the compactness of the test group (No. 2–4) was lower than that of the CK (No. 1) (by 14.3–28.3%).

### 4.2. Organic Matter Content

**Figure 4:**
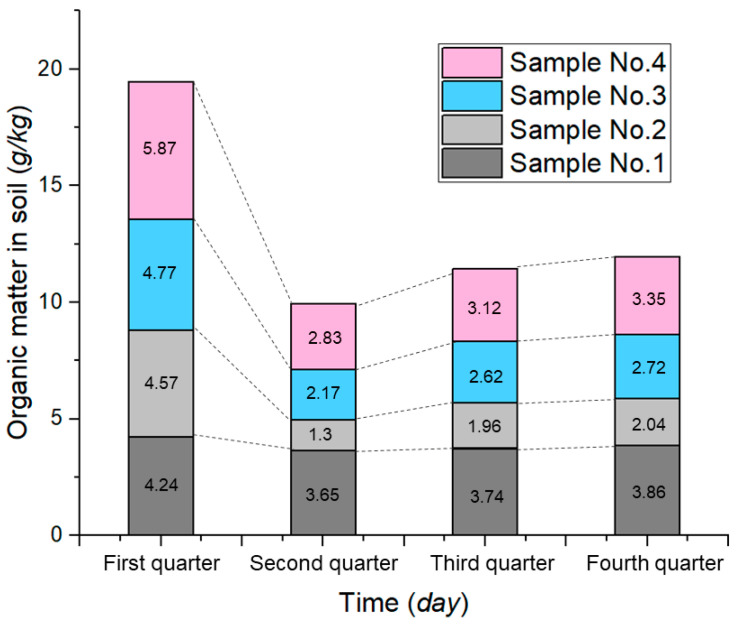
Organic matter content of soil.

Soil organic matter content is one of the important indexes of soil fertility, and increasing soil organic matter content can improve the soil fertility [33,34]. The organic matter content in the soil is shown in Figure 4. In the first quarter, the organic matter content of all groups was high and increased with the increase in material content. In the second quarter, the organic matter content of the soil decreased in different degrees, and compared with the first quarter, the organic matter content of No. 1–4 decreased by 13.92%, 71.55%, 54.51%, and 51.79%, respectively. In the third quarter, the organic matter content of the soil increased in all groups, and compared with the second quarter, the organic matter content of No. 1–4 is increased by 2.47%, 50.77%, 20.74%, and 10.25%, respectively. In the fourth quarter, the organic matter content of soil in all groups continued to increase slowly; compared with the third quarter, the organic matter of No. 1–4 increased by 3.21%, 4.08%, 3.82%, and 7.37%, respectively.

### 4.3. Total and Available Nutrient Content of the Soil

Nutrients in soils are classified into total and available nutrients according to the degree of difficulty in their utilization by plants [35]. Total nutrients are the total amount of all forms of nutrients (*n*, *p*, K) in the soil; available nutrients are generally water-soluble and exchangeable nutrients in the soil, which can be directly absorbed and used by plants or can be quickly exchanged from the soil colloid for plant use.

At the early stage of the test, the total nutrients in the soil samples of each group were measured, and the results are shown in Table 3. The total nutrient contents of each group were very similar, indicating that the initial conditions of the tested soils were basically the same in each group. This is because the test soils were taken from the same location and were uniformly air-dried, milled, and sieved (particle size <2 mm) before being mixed for use.

**Figure 5:**
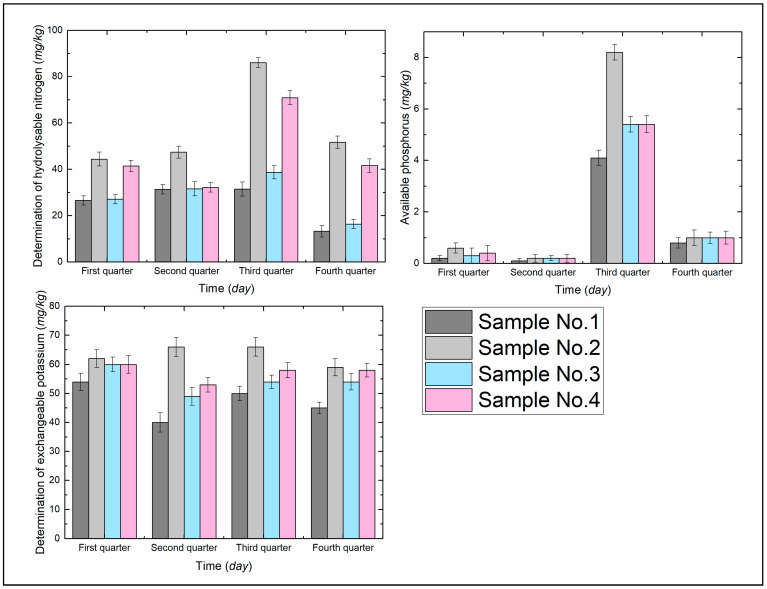
Available nutrients content of the soil.

Inorganic nutrients from the conversion and decomposition of organic matter are an important source of available nutrients for the soil and play an important role in plant growth [34]. Samples were collected in the first, second, third, and fourth quarters to measure the available nutrients of the soil. As can be seen from Figure 5, the content of available nitrogen, phosphorus, and potassium in the soil in all periods showed a trend of No. 2 > No. 4 > No. 3 > No. 1.

Compared with No. 1, the available nitrogen content of No. 2–4 increased by 66.92%, 1.88%, and 55.64%, respectively, in the first quarter; 50.96%, 0.64%, and 2.55%, respectively, in the second quarter; 173.02%, 22.86%, and 125.08%, respectively, in the third quarter; and 288.72%, 23.31%, and 212.78%, respectively, in the fourth quarter.

Compared with No. 1, the available phosphorus content of No. 2–4 increased by 200%, 50%, and 100%, respectively, in the firstly quarter; 100%, 100%, and 100%, respectively, in the second quarter; 100%, 31.71%, and 31.71%, respectively, in the third quarter; and 25%, 25%, and 25%, respectively, in the fourth quarter.

Compared with No. 1, the available potassium content of No. 2–4 increased by 14.81%, 11.11%, and 11.11%, respectively, in the firstly quarter; 65%, 22.5%, and 32.5%, respectively, in the second quarter; 32%, 8%, and 16%, respectively, in the third quarter; and 31.11%, 20%, and 28.89% respectively, in the fourth quarter.

### 4.4. Microorganism Content of the Soil

As an important component of the biochemical cycle in terrestrial ecosystems, soil microorganisms are important in the conversion and decomposition of soil organic matter, nutrient formation, and uptake, and are sensitive indicators of changes in the soil environment [36].

**Figure 6:**
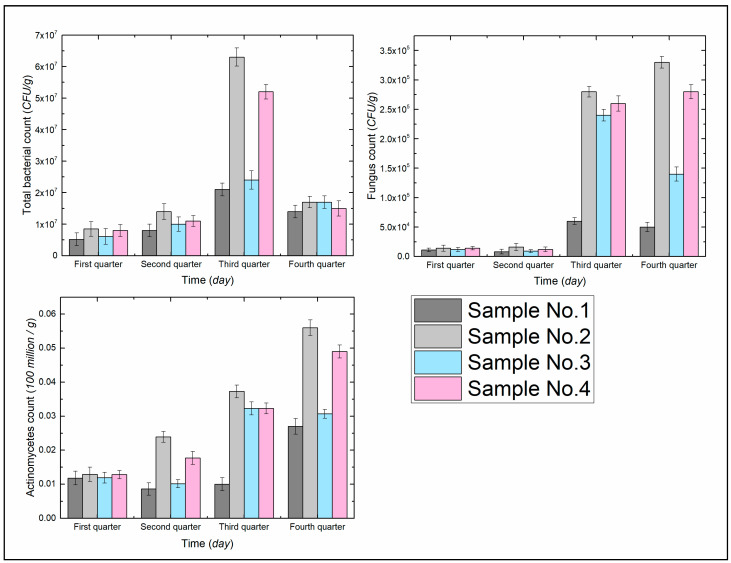
Number of microorganisms in the soil.

As shown in Figure 6, in the first quarter, the number of microorganisms in the soil was small. Compared with No. 1, the number of microorganisms in No. 2–4 increased to different degrees, among which the number of bacteria increased by 63.46%, 17.31%, and 53.85%, in order; the number of fungi increased by 27.27%, 9.09%, and 27.27%, in order; the number of actinomycetes numbers increased by 9.32%, 0.85%, and 8.47%, in that order.

Compared with the first quarter, the number of microorganisms changed very little in the second quarter; the number of bacteria in No. 1–4 increased by 53.85%, 64.71%, 63.93%, and 37.5%; the number of fungi increased by −25.45%, 14.29%, −24.17%, and −14.28%; and the number of actinomycetes increased by −27.37%, 85.27%, −15.13%, and 38.28%, respectively.

Compared with the second quarter, the number of microorganisms in each group increased significantly in the third quarter. The number of bacteria in No. 1–4 increased by 162.5%, 350%, 140%, and 372.7%; the number of fungi increased by 631.71%, 1650%, 2537.36%, and 2066.67%; the number of actinomycetes increased by 16.34%, 56.07%, 219.80%, and 82.49%, respectively.

Compared with the third quarter, the number of bacteria decreased in all groups in the fourth quarter, and there was no significant trend in the number of fungi and actinomycetes. The number of bacteria in No. 1–4 increased by −33.33%, −73.02%, −29.17%, and −71.15%, in that order; the number of fungi increased by −16.67%, 17.86%, −41.67%, and 7.69%, in that order; and the number of actinomycetes improved by 170.81%, 50.13%, −4.95% and 51.70%, in that order.

### 4.5. Plant Growth Effect

#### 4.5.1. Germination Rate, Germination Time, and Growth Curve

Table 4 compares the germination time and rate of each group of samples. Compared with No. 1, the germination time of No. 2–4 was two days earlier, and the germination rate increased by 40%, 20%, and 34% in that order.

**Figure 7:**
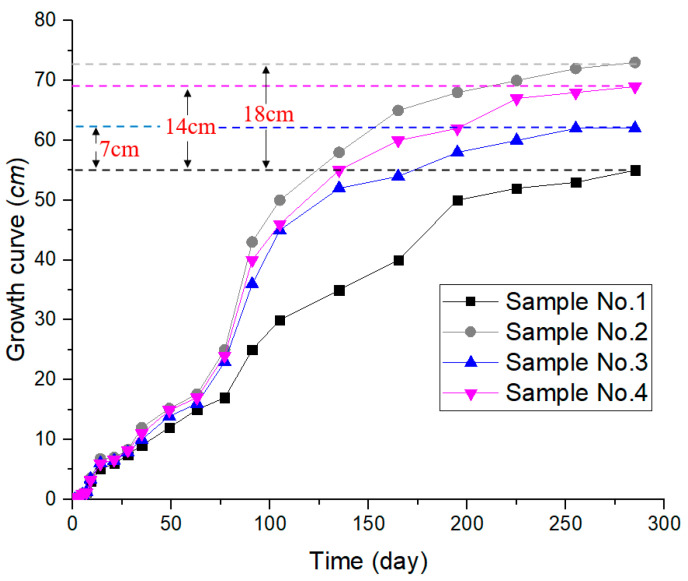
Plant growth curve.

After the plants germinated, the height of the plants was measured manually. Where plant height is defined as the distance from the surface of the soil to the top of the plant, the results are shown in Figure 7. The plants of No. 2 grew the best and the No. 1 grew the worst. On the last day, for example, the plant heights of No. 2–4 increased by 32.73%, 12.73%, and 25.45%, respectively, compared to No. 1.

#### 4.5.2. Plant Coverage Rate

**Figure 8:**
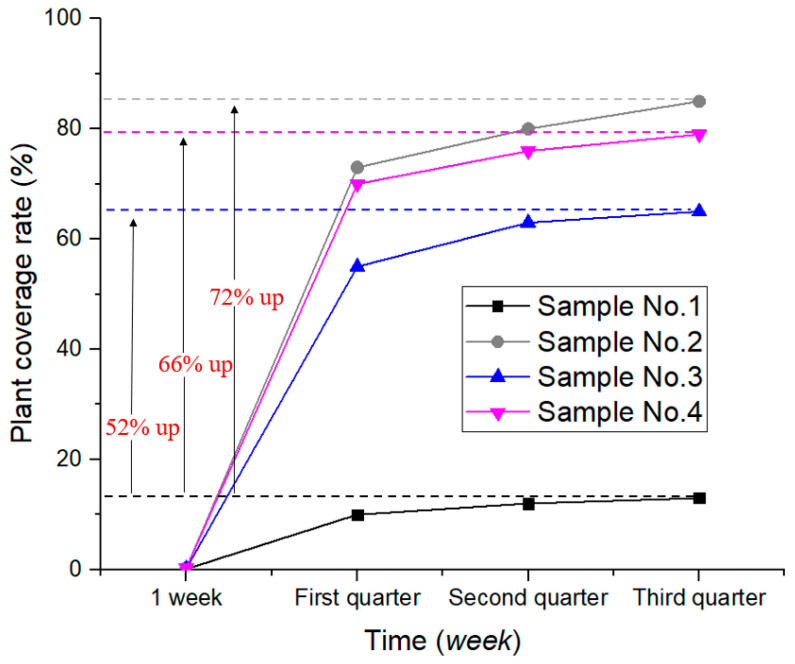
Plant coverage rate.

Plant coverage rate was defined as the percentage of the vertical projection of plant (including leaves, stems, and branches) on the ground to the total area of the statistical area. The plant coverage of each group is shown in Figure 8. No. 2 has the highest coverage and No. 1 has the lowest. As an example, the coverage rate of No. 2–4 increased by 553.85%, 400%, and 507.69%, respectively, compared with No. 1 in the fourth quarter.

**Figure 9:**
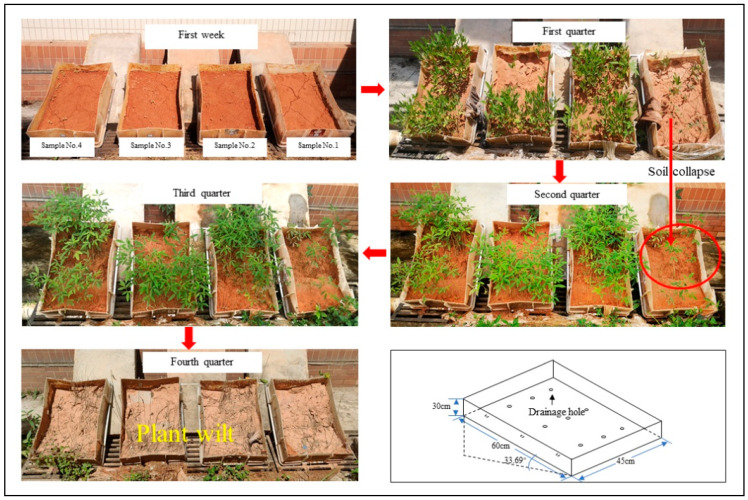
Plant growth effect.

The growth of the plants at different times of the year is shown in Figure 9. It can be seen that: ADNB can effectively improve the germination rate of seeds and promote plant growth, but the ratio of ADNB will affect the final effect, and a reasonable ratio can achieve the best results. Therefore, the actual use needs to be blended according to the local climate, soil, and engineering conditions.

## 5. Discussion

### 5.1. Effect of ADNB on Compactness

Soil compactness, as one of the main physical properties of the soil, is influenced by factors such as mechanical composition, organic matter content, and soil moisture. Large soil compactness can cause difficulties with water infiltration, fertilizer nutrient utilization, and root growth inhibition [37], while smaller soil compactness can lead to problems such as loss of soil stability on slopes and erosion vulnerability. Relevant studies found that a reasonable soil compactness is conducive to enhancing the reinforcing effect of plant roots [38]. So, keeping the compactness of soil in a reasonable range has a positive effect on the ecological restoration of slopes. The NAB in ADNB can improve the initial strength of soil by increasing the cohesion and internal friction angle between soil particles [20], and for SAR in ADNB as a resin-like material, it swells in water absorption and shrinks in water loss, and the repeated changes of SAR volume will destroy the original skeleton structure of the soil, making the soil loose and avoiding later consolidation [19].

From the technical documentation provided by the manufacturer of the compactness meter (STEPS, Germany) [22], most plant roots can grow well when the soil compactness is less than 200 psi, average at 200 psi–300 psi, and difficult at compactness greater than 300 psi. It can be seen from Figure 3 that after one year under natural conditions, the soil of the CK (No. 1) has become consolidation, which is not conducive to the growth and development of plants in the coming year. In contrast, the compactness of the test group (No. 2–4) has been kept in a reasonable range which can meet the soil strength requirements without hindering the growth of plant roots and enhance the stability of the slope soil from both the soil and vegetation aspects.

### 5.2. Effect of ADNB on Organic Matter

The test soils were adequately air-dried, milled, and mixed at the test preparation stage, so that the initial organic matter content of each group of soil samples was basically the same. In the first quarter, the organic matter content of each group increased with the increase in ADNB content. This is due to the fact that both materials in the ADNB are polymeric organic substances, and they are counted as soil organic matter when measurements are made using the potassium dichromate capacity method [23]. During this period, the difference in the organic matter content of the soil depends on the amount of ADNB content. With the passage of time, the organic matter content of each group in the second quarter decreased, and No. 1 organic matter decreased the least and No. 2 decreased the most. Combined with the plant growth date (Figure 7), it can be seen that plant growth consumed a large amount of soil organic matter, resulting in different degrees of organic matter decrease. By the third and fourth quarter, the organic matter of all group slowly increased, which could be attributed to the decomposition of organic matter fragments, ungerminated seeds, and fallen leaves originally contained in the soil to form new organic matter.

### 5.3. Effect of ADNB on Soil Nutrients and Microorganisms

There are two possible reasons for the slight differences in the total nutrients in the soil: (1) ADNB contains a small number of related elements; (2) NAA in the ADNB is slightly acidic, and spraying it on the surface of the soil will accelerate the release of elements stored in the mineral crystals.

It can be seen by Figure 5 and Figure 6 that ADNB can increase the available nutrients and number of microorganisms in the soil, and is higher than the natural soil (CK) in all periods. The improvement of the organic matter content, soil structure [19], and soil–water characteristics relationship [21] by ADNB is the main reason for the increase in nutrient level and microorganism population in the soil. On the one hand, ADNB, as a polymer organic compound, can be regarded as an additional organic matter input [15], providing nutrients to the soil; on the other hand, loose soil structure, reasonable water content, and sufficient nutrient supply provide good conditions for microbial metabolism and reproduction, which is conducive to the role of microorganisms in improving soil fertility. The two factors mentioned above work together to create a good growth environment for plants, and along with plant growth, plant root secretions gradually increase, which can further improve the microbial survival environment and provide carbon and energy sources for microbial reproduction and metabolism [23].

Bacterial populations decreased significantly in the fourth quarter, probably due to the deterioration of their living environment caused by soil drying and plant wilting, and their greater ability to utilize water-soluble compounds compared to fungi and actinomycetes. Therefore, they are also most affected by the environment and are the first to show a decrease in numbers.

### 5.4. Effect of ADNB on Plant Growth

Comparing the growth of plants in the test group (No. 2–4) and the control group (No. 1) under uniform water and fertilizer management, it can be seen (Figure 7, Figure 8 and Figure 9) that the germination rate and germination time of the ADNB-treated samples were higher than those of the natural soil samples, and the growth curve of the test group (No. 2–4) was consistently higher than that of the control group. It can be seen that a suitable growth environment, sufficient nutrient supply, and stronger microorganism activity can effectively promote the germination and growth of plants. The ecological self-repairing ability of surface slope soil can be effectively enhanced by adding new polymeric materials (ADNB) to the soil.

### 5.5. Effect of Material Ratio on Improvement Effect

The plant growth of ADNB-treated samples (No.2–4) was significantly better than that of natural soil (No.1), but the plant growth effect varied with different ratios of ADNB. The plant growth of No. 2 and No. 4 was significantly better than that of No. 3, and in the No. 3 sample, the plants were mainly distributed in the lower half, which was because the percentage of SAR in No. 3 (SAR mass/total mass of ADNB) is higher and the larger amount of SAR expansion destroys the surface curing layer, resulting in reduced stability of the improved soil, and rainfall erosion washes the seeds from the upper part to the lower part, which affects the growth of plants. It can also be seen by Figure 9 that after one year, the surface integrity of No. 2 and No. 4 was better compared to No. 1 and No. 3, indicating that reasonable ADNB ratios can achieve longer-term protection.

## 6. Mechanistic Analysis

### 6.1. Soil Improvement Mechanism

The nano-aqueous binder (NAB) in ADNB can increase the degree of soil particle aggregation through adsorption and bonding, and form a solidified layer on the soil surface to enhance the ability of soil particles to resist relative displacement between particles and improve the strength and stability of the soil surface. When soil moisture is sufficient, the super absorption resin (SAR) in ADNB will adsorb the excess free water and store it in its own molecular structure; when soil moisture decreases, the water in the resin will be released slowly under the action of substrate suction and supplied to plant growth. The volume change of the SAR in the process of water absorption and release plays the role of loosening the soil and increasing the porosity and permeability of the soil.

**Figure 10:**
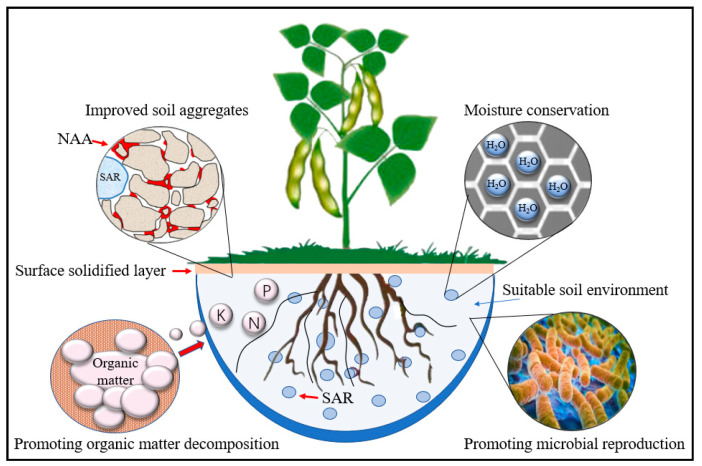
Schematic diagram of the mechanism of ADNB-improved soil.

Under the combined action of the NAB and SAR, the soil structure was optimized; the compactness [20], water holding capacity, and water supply capacity [21] of the soil were improved to different degrees; and the increase in water content also increased the specific heat capacity of the soil and reduced the diurnal temperature difference, thus achieving the effect of improving the plant living environment [19]. The improvement of living environment is conducive to the reproduction of microorganisms, and a large number of microbial activities can effectively improve the decomposition efficiency of organic matter, thus increasing the nutrient supply capacity of the soil and promoting plant growth; while in the process of plant growth, plant roots continuously secrete large amounts of organic matter into the soil, forming inter-root deposits, which provide abundant nutrients and energy for the growth of microorganisms [39]. The dynamics of the microbial community are closely related to the growth of plants [40]. In summary, ADNB improves the nutrient supply capacity of the soil by improving the physical properties of the soil, creating a good growth environment for plants and microorganisms and ultimately enhancing the ecological self-repairing ability of surface slope soil (Figure 10).

### 6.2. The Long-Term Effect

**Figure 11:**
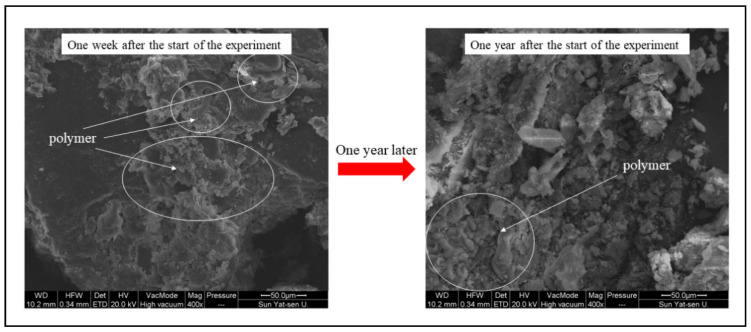
Electron microscope scan image.

To investigate the adsorption and aggregation effect of ADNB on soil particles and its degradation phenomenon in natural environment, the microstructure of soil samples at the beginning of the test and after one year was compared using SEM. From Figure 11, it can be seen that the NAB in ADNB can effectively adsorb and aggregate soil particles, thus improving the soil structure. However, it will gradually degrade under a natural environment, and the content of NAB in the soil decreased to a certain degree after one year, but some of it was still maintained. According to the previous research of our team [20,21], the degradation period of NAB and SAR in the natural environment is 24–36 months, which can cover two to three growth periods of plants, providing sufficient growth time for vegetation community growth and natural succession.

## 7. Conclusions

Based on outdoor model tests, this paper investigates the effects of different ADNB ratios on soil compactness, biochemical properties, and plant growth at longer time scales and explores its effects and mechanisms on the enhanced ecological self-repairing ability of surface slope soils. The conclusions are as follows:

(1) A new polymer composite material (ADNB) was compounded with a polymer nano-aqueous binder (NAB) and super absorption resin (SAR) for improving the surface soil of slopes. The results show that ADNB can not only improve the stability of slopes by increasing the compactness of soil, but also optimize the water and fertilizer supply capacity of soil by increasing the amount of organic matter, available nutrients, and microorganisms in soil.
(2) Outdoor model tests were conducted based on the new polymer composite material (ANDB), and it was found that ADNB can enhance the ecological self-repairing ability of surface slope soil by improving soil structure, reducing soil erosion, and optimizing water and fertilizer supply, thus promoting plant growth; the degradation period is as long as 24–36 months, which can completely cover two to three growth periods of plants and can provide sufficient growth time for the growth and natural evolution of vegetation communities.
(3) ADNB needs to select an optimal proportioning scheme according to the actual situation, taking into account the local soil quality, climate, rainfall, etc., so as to meet the stability requirements while controlling the looseness of the soil in an appropriate range to achieve the optimal improvement effect.

## Figures and Tables

**Table 1:** Physical and mechanical parameters of soil.

Parameter of Natural State	Natural density ρ_o_	1.98 g/cm^3^
	Specific gravity Gs	2.68
	Water content ω	19.7%
	Void ratio e	0.62
	Degree of saturation Sr	85%
Index of Consistency	Liquid limit ωL	30.2%
	Plasticity limit ωp	18.2%
	Liquidity inde I_L_	0.13
	Plasticity inde I_p_	12.0
Index of Consolidation	Compressibility α_V_	0.338 MPa^−1^
	Compression modulus Es	4.79 MPa
Mechanical Parameters	Cohesion *C*	34 kPa
	Internal friction angle φ	25.9°

**Table 2:** Material ratio.

Material Type	No. 1 (CK)	No. 2	No. 3	No. 4
NAB (g/m^2^)	0	10	10	15
SAR (g/m^2^)	0	60	70	70

**Table 3:** Total nutrient content of the soil.

Type	No. 1	No. 2	No. 3	No. 4
Total nitrogen (%)	0.029	0.031	0.030	0.031
Total phosphorus (%)	0.037	0.037	0.037	0.036
Total potassium (%)	2.54	2.59	2.54	2.57

**Table 4:** Plant germination time and rate.

Type	Germination Time (Day)	Germination Rate (%)
No. 1	5	15
No. 2	3	55
No. 3		35
No. 4		49

## Data Availability

Not applicable.
